# Causes and Prevention of Early-Onset Colorectal Cancer

**DOI:** 10.7759/cureus.45095

**Published:** 2023-09-12

**Authors:** Aisha O Adigun, Temitayo M Adebile, Chiugo Okoye, Taiwo I Ogundipe, Omolola R Ajekigbe, Rheiner N Mbaezue, Okelue E Okobi

**Affiliations:** 1 Infectious Diseases, University of Louisville, Louisville, USA; 2 Public Health, Georgia Southern University, Statesboro, USA; 3 Nephrology, Boston Medical Center, Boston, USA; 4 Internal Medicine, California Institute of Behavioral Neurosciences & Psychology, Fairfield, USA; 5 Internal Medicine, Palm Coast Urgent Care, Orlando, USA; 6 Family Medicine, Ladoke Akintola University of Technology, Ogbomoso, NGA; 7 Health Services, Department of Health, Cape Town, ZAF; 8 Family Medicine, Larkin Community Hospital Palm Springs Campus, Hialeah, USA; 9 Family Medicine, Medficient Health Systems, Laurel, USA; 10 Family Medicine, Lakeside Medical Center, Belle Glade, USA

**Keywords:** diet, lifestyle, risk factors, prevention, colorectal cancer, sporadic, early-onset

## Abstract

Sporadic colorectal cancer (CRC) has historically been considered a disease of the elderly. However, early-onset colorectal cancer (eoCRC) incidence and prevalence have steadily increased over the last few decades, highlighting the critical need for a comprehensive understanding of its causes and prevention. This research examines the numerous factors contributing to the increasing incidence of eoCRC. These factors include a combination of genetic predispositions and environmental effects. We also investigate the impact of modifiable lifestyle factors like obesity, physical inactivity, and an unhealthy diet on eoCRC risk. Understanding these factors is critical in developing future diagnostic, prognostic, disease monitoring, and therapy planning strategies in managing eoCRC and will help optimize guidelines for CRC screening.

## Introduction and background

Colorectal cancer (CRC) is a serious public health concern worldwide, causing significant morbidity and mortality. Traditionally considered a disease affecting older adults, early-onset colorectal cancer (eoCRC) has recently seen an alarming increase among younger individuals [1]. CRC develops due to the malignant alteration of colon or rectal epithelial cells. It is the third most frequent cancer in the world and ranks as the second leading cause of cancer-related death [1]. The disease occurs due to a complex interaction of genetic, environmental, and lifestyle factors, with the risk increasing with age. A family history of CRC-inherited genetic mutations, inflammatory bowel disease, obesity, sedentary lifestyle, smoking, heavy alcohol consumption, and certain dietary habits are all common risk factors for CRC [2-4].

Recently, a concerning trend in the incidence of CRC in young adults has emerged. While overall rates of CRC in people over 50 years have decreased [5], there has been a continuous increase in the prevalence of eoCRC, defined as a CRC diagnosis in someone under 50 years old. Multiple countries have reported increased eoCRC cases globally, indicating additional research and preventive actions are needed. The United States has also witnessed a particularly significant increase in eoCRC. A study by the American Cancer Society found that the proportion of cases among individuals aged 20-54 years increased from 11% in 1995 to 20% in 2019. Between 2011 and 2020, colon cancer mortality increased by 0.5%-3% annually in individuals below 50 years [1].

Understanding the causes and prevention of eoCRC is necessary for various reasons. First, the increased incidence of eoCRC has significant public health implications because it impacts the lives of the affected individuals and causes an enormous strain on healthcare systems. In addition, eoCRC is detected at a later stage and has distinct clinical and molecular characteristics compared to late-onset CRC [2], emphasizing the need for specialized research and preventative methods specific to this age group.

This study aims to investigate the causes and prevention of eoCRC thoroughly. By combining and assessing existing research findings, we aim to uncover the critical factors linked with the development of eoCRC and potential interventions that could help reduce its incidence. This evaluation will also shed light on present knowledge gaps and give a road map for future research efforts.

## Review

Screening and early detection

Early detection and timely diagnosis of eoCRC are crucial for better disease outcomes. To address the increasing incidence of CRC among young adults, the American Cancer Society (ACS) and the US Preventive Services Task Force (USPSTF) have updated their screening guidelines. They now recommend regular screening for CRC for all adults with an average risk starting from age 45 [6]. The guidelines suggest that screening decisions should consider the balance between the benefits and burdens of screening and patient preferences [7]. Screening is recommended until age 75 for healthy adults with average risk and a life expectancy greater than 10 years. Beyond age 85, screening for CRC is discouraged. Individuals with a first-degree relative with CRC may start screening at the age of 40 or 10 years before the age of diagnosis of the youngest affected relative. Those with second-degree relatives with CRC or polyps may begin screening at age 40 [8]. The ACS and the USPSTF recommend several screening options for CRC. Stool-based tests include a stool DNA test every three years, an annual fecal immunochemical test (FIT), and a yearly guaiac-based fecal occult blood test (gFOBT). Direct visualization tests include colonoscopy every 10 years, CT colonography every five years, and flexible sigmoidoscopy every five years. If flexible sigmoidoscopy or CT colonography results are abnormal, further evaluation with a colonoscopy is recommended [7,8].

Challenges With Early Detection in Young Adults

Despite the climbing rate of eoCRC and the survival benefit of early detection, screening rates remain low among young adults. Early cancer detection among young adults poses a challenge than in other age groups. Chen et al. discovered seven weeks to two years of detection delay among patients under 50 compared to those aged 50 years and older [9]. In this retrospective study among 485 patients with colorectal adenocarcinoma, patients under 50 years had a longer median time to detection (128 days) compared to patients 50 years and older (79 days). Also, according to the results obtained from their multivariable analysis, diagnosis time was 1.4 times longer for patients less than 50 years old compared to those 50 years and above.

A plethora of studies [8-10] have explored the barriers to the rate of CRC screening uptake. In the same study by Chen et al., the authors found a positive correlation between the duration of symptoms and time to diagnosis in their younger cohort. They argued that the delay in detection could be attributed to younger individuals ascribing lower priority to their symptoms [9]. The absence of provider referral was a barrier to undergoing CRC screening in two studies [8,10]. Physicians may assume a benign diagnosis because of a low level of suspicion for CRC and delay referral for a diagnostic colonoscopy. In Myers et al.'s study, some patients who presented with intermittent rectal bleeding were treated for hemorrhoids for over 12 months before they were sent for colonoscopy [8]. Other identified barriers to CRC screening include fear and worry, financial issues like lack of insurance, screening cost, logistics surrounding screening, and problems with bowel preparation [10].

While there may be many benefits to lowering the screening age to 45 years and older, the question of diverting resources from higher-risk older individuals to lower-risk, younger individuals remains. While the incidence of CRC has increased among individuals below age 50, the rate of CRC among younger adults cannot be compared to the increase in CRC among individuals 50 years and above. Likewise, the mortality rate of CRC among individuals aged 50 years and older has been estimated to be two to six times that of individuals aged 45-49 years [11]. Ladabaum et al. compared the cost-effectiveness of screening adults aged 45 to 75 years with adults aged 50 to 75 years and learned that starting CRC screening at age 45 years is potentially more cost-effective compared to starting at 50 years. Nonetheless, the results of their sensitivity analysis imply that focusing on older and higher-risk persons instead of average-risk 45-year-olds would yield substantial net savings. They argued that much societal benefit could be derived by improving screening rates among older and higher-risk persons or ensuring an abnormal FIT result is followed up with a colonoscopy [11].

Importance of Early Detection and Screening in Reducing Incidence and Mortality

Early CRC detection among young adults will potentially save lives since precancerous lesions and early stages of CRC will be caught early. Studies among older adults have previously shown the association of CRC screening with a reduction in the incidence of CRC and CRC-related deaths. These findings may be applicable to young adults. For instance, Doubeni et al. conducted a nested, large, community-based case-control study among 1747 patients who died from CRC and 3460 CRC-free controls to determine if colonoscopy screening reduced mortality among average-risk individuals. They found a 67% reduction (adjusted OR (aOR) = 0.33, 95% CI = 0.21 to 0.52) in the risk of death from any CRC among those who had a screening colonoscopy compared to those who did not receive a screening colonoscopy [12].

Additionally, CRC screening was associated with a significant decrease in the incidence of and mortality from CRC in a study by Schoen et al., who evaluated the effect of screening with flexible sigmoidoscopy on CRC incidence and mortality [13]. They randomly assigned 154,900 participants to undergo CRC screening with flexible sigmoidoscopy or to usual care. After 11.9 years, the authors observed a 21% reduction (RR = 0.79; 95% CI = 0.72 to 0.85; P < 0.001) in the incidence of CRC among the participants in the screening/intervention group compared to the usual-care group. Again, they saw a 26% reduction (relative risk, 0.74; 95% CI, 0.63 to 0.87; P < 0.001) in CRC mortality among the screening group participants compared to the usual-care group. Their conclusions further indicate that the reduction in mortality after colonoscopy may depend on the location of cancer in the colon because they observed a 50% reduction in mortality from distal CRC compared to the unchanged mortality rate of proximal CRC. The location-dependent decrease in mortality of CRC was established in a study conducted a couple of years earlier among a Canadian population by Singh et al. [14]. Although the authors saw a 29% reduction in CRC mortality (standardized mortality ratio, 0.71; 95% CI, 0.61-0.82) among the participants who underwent screening colonoscopy, compared to the 47% reduction in mortality from distal CRC, they also observed no reduction in mortality from proximal CRC [14].

Factors affecting the development of eoCRC

Familial and Genetic Factors

The rise in the incidence of eoCRC is increasingly alarming and necessitates an in-depth understanding of its etiology, particularly the role of genetic (the role of an individual's genetic makeup in determining their susceptibility to CRC) and familial (cases of CRC that occur more frequently within a family than would be expected by chance alone) risk factors. Genetic factors significantly contribute, with Lynch syndrome (LS) [15] and familial adenomatous polyposis (FAP) [16] being the most common inherited CRC syndromes. LS, resulting from germline mutations in DNA mismatch repair (MMR) genes, is associated with up to 80% lifetime CRC risk, often presenting with microsatellite instability (MSI) [17]. FAP, resulting from mutations in the adenomatous polyposis coli (APC) gene, is characterized by early-onset adenomatous polyps, leading to nearly 100% lifetime CRC risk if untreated [18].

Furthermore, several other genetic syndromes, such as MutYH-associated polyposis (MAP) [19], Peutz-Jeghers syndrome, juvenile polyposis syndrome, and Li-Fraumeni syndrome, are associated with an increased risk of eoCRC [20]. Mutations in BRCA1 and BRCA2 genes, traditionally linked with breast and ovarian cancers, have also been implicated [21]. Somatic mutations, notably in tumor protein 53 (TP53), Kirsten rat sarcoma viral oncogene homolog (KRAS), and APC genes, are also common in eoCRC, suggesting potential pathogenesis pathways [22].

**Figure 1:**
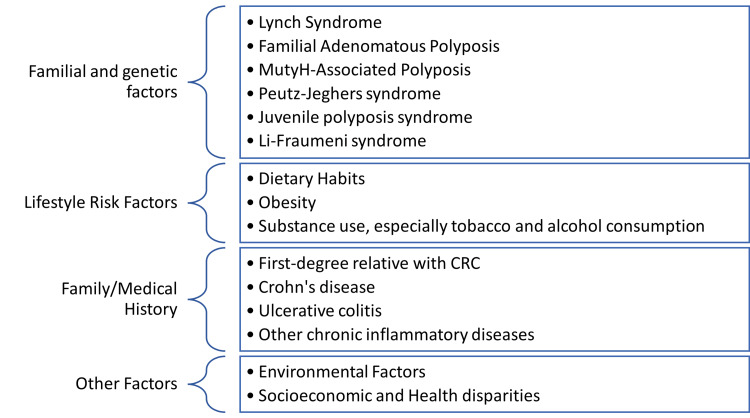
An original diagram showing common risk factors of early-onset colorectal cancer. CRC: colorectal cancer.

Although most studies support the theory that having a first-degree relative with CRC considerably increases the risk, especially if diagnosed at a young age, the research is contradictory. A cross-sectional study of the increased incidence of eoCRC in the Egyptian population by Abou-Zeid et al. found that family history criteria and pathologic features of tumors in young Egyptian patients do not differ significantly from those in older patients, implying that they have a similar etiology. Hereditary risk factors are unlikely to be the origin of this atypical pattern of eoCRC in Egyptian patients [23]. Figure 1 below illustrates the most common factors associated with the development of eoCRC.

Lifestyle Factors

Many eoCRC cases are attributed to various lifestyle factors, including obesity, dietary habits, and substance use. Obesity has emerged as a prominent risk factor for eoCRC, with numerous studies highlighting a significant association [24]. A large prospective study of women by Liu et al. revealed that a higher current BMI at 18 and weight gain since early adulthood were associated with an increased risk of eoCRC [3]. This increased incidence is likely due to chronic inflammation, metabolic syndrome, and insulin resistance promoting carcinogenesis [25]. Obesity plays a role in the development of colon cancer by altering the composition of gut bacteria [26]. This change leads to higher levels of inflammation-causing molecules (such as lipopolysaccharide, increased acetate, and reduced butyrate) that can weaken the intestinal barrier [27]. Other harmful effects caused by this imbalance in gut bacteria include epigenetic changes that affect gene function and changes in the byproducts produced by gut bacteria. Increased deoxycholic acid, a secondary bile acid from certain bacteria, can cause DNA damage [28]. The evidence suggests that decreasing body weight could potentially influence the incidence of colon cancer, given its positive effects on reducing chronic inflammation and insulin resistance.

Dietary habits also play a pivotal role in the development of eoCRC. A Western-style diet characterized by a high intake of red and processed meats, refined grains, and sugary beverages and low in fruits, vegetables, and whole grains has been implicated in increasing CRC risk [29]. In contrast, diets rich in fiber, such as the Mediterranean diet, are associated with reduced risk, potentially due to their influence on the gut microbiota and reduced pro-inflammatory compounds [30]. A case-control study by Puzzono et al. to evaluate the diet and lifestyle habits of individuals with eoCRC, compared to age-matched healthy controls, determined that processed meat, dairy products, and smoking could be considered significant risk factors for eoCRC [31]. Multiple other studies studying the relationship between diet, smoking, and alcohol intake [32,33] on the development of eoCRC also came to the same conclusion. According to the American Cancer Center, eating a lot of red and processed meat can harm and promote growth in the intestinal lining. This can lead to colon cancer due to DNA damage caused by toxic compounds like heterocyclic amines and polycyclic aromatic hydrocarbons (PAH). Red and processed meats contain several added and naturally occurring chemicals that can cause cancer. For instance, Haem, a naturally occurring chemical in red meat, breaks down in the intestines and causes a process known as lipid peroxidation. This leads to the creation of N-nitroso chemicals, which are harmful. These N-nitroso chemicals can also be found in processed meats because they are added as preservatives, nitrites, or nitrates. Substituting red and processed meats, foods high in saturated fats, refined starches, and sugars with fish, poultry, vegetables, fruits, and grains as sources of polyunsaturated fats, carbohydrates, and proteins could potentially reduce the risk of developing eoCRC [30].

Substance use, especially tobacco and alcohol consumption, contributes significantly to the risk of eoCRC. Smoking has been linked to an increased risk of eoCRC, with the risk escalating with the duration and intensity of smoking [34]. This increased risk is thought to be a result of the aromatic amines, nitrosamines, heterocyclic amines, and polycyclic aromatic hydrocarbons present in cigarette smoke. Meanwhile, alcohol intake ≥ 20 g/day [33] has been recognized as a risk factor for eoCRC, possibly due to the carcinogenic effects of acetaldehyde, a metabolic product of ethanol, a class 1 carcinogen known to cause damage to chromosomes [32]. These lifestyle-related risk factors, coupled with increasing sedentary behavior, underline the importance of targeted public health interventions focusing on lifestyle modifications to curb the rising trend of eoCRC. Based on the evidence in the literature, it is safe to assume that smoking and alcohol cessation can significantly reduce the risk of eoCRC.

Environmental Factors

Based on emerging data, certain environmental exposures may have a substantial role in the development of eoCRC [35]. Exposure to environmental contaminants is one such factor. According to research, long-term exposure to pollutants such as fine particulate matter (PM2.5) and toxic metals may contribute to colorectal carcinogenesis by creating reactive oxygen species (ROS) [36] and leading to systemic inflammation [35].

Radiation exposure, whether through occupational exposure or medical treatments, has also been linked to an increased risk of eoCRC. Ionizing radiation can directly damage DNA or create ROS that cause DNA mutations, promoting tumors. This risk factor is significant for people who have had radiation therapy from prior cancers, especially during childhood or adolescence, because they are more likely to develop secondary malignancies, including CRC [37].

Another environmental factor is the influence of the built environment on lifestyle behaviors. Urbanization and resultant lifestyle changes, such as decreased physical activity due to less active commuting and increased intake of processed foods, contribute indirectly to the risk of eoCRC [38]. These factors, often associated with sedentariness and obesity, play a crucial role in colorectal carcinogenesis. Therefore, understanding the broader environmental context is critical in devising effective preventive strategies against eoCRC. However, more research is needed to fully elucidate the complex interplay between environmental exposures and genetic and lifestyle factors in the pathogenesis of eoCRC.

Medical History

Studies have shown that certain aspects of a person's medical history can considerably raise their risk of eoCRC. A history of chronic inflammatory bowel diseases (IBD), such as Crohn's disease and ulcerative colitis, is at the top of the list [39]. According to a large retrospective study, individuals with IBD risk developing eoCRC more than those without the condition [2]. This risk rises with disease duration, the extent of colon involvement, and additional conditions such as primary sclerosing cholangitis [40].

Certain metabolic disorders, such as type 2 diabetes and insulin resistance, have also been implicated in the increased risk of eoCRC [41]. Shared risk factors such as obesity and a sedentary lifestyle also contribute to this elevated risk. Although the exact mechanisms are still under investigation, it is proposed that hyperinsulinemia and hyperglycemia may promote the growth and proliferation of colonic epithelial cells, thereby leading to tumorigenesis [41]. While many risk factors for eoCRC are not modifiable, understanding the role of medical history can help inform early detection strategies and risk reduction interventions in susceptible individuals.

Socioeconomic and Health Disparities

Socioeconomic and health disparities remain among the most significant factors influencing eoCRC risk and outcomes. These disparities, rooted in complex social stratification systems, affect cancer incidence and mortality, disease stage at diagnosis, and access to quality care.

Low socioeconomic status (SES) is associated with higher eoCRC incidence and poorer survival outcomes. Individuals in the lower SES strata often face barriers to healthcare access, including CRC screening, resulting in later-stage diagnoses and worse prognoses [9,42]. Additionally, lifestyle factors associated with low SES, such as poor diet, physical inactivity, and higher rates of tobacco and alcohol use, may further increase the risk of eoCRC. The observed socioeconomic differences in CRC may not necessarily hold for early-onset cases. This is due to the potential differences in biological mechanisms and the possible lower relevance of screening access for those with early disease onset. Health insurance status is another aspect of health disparities affecting eoCRC. Uninsured and underinsured individuals are more likely to be diagnosed with advanced-stage disease and have lower survival rates, mainly due to barriers to accessing preventive services like screening and early detection [43].

Racial and ethnic disparities also significantly influence eoCRC incidence and outcomes. In the United States, for instance, Asian and Black populations have the highest incidence and lowest survival rates of CRC across all age groups, including young adults. A study by Myers et al. found a higher incidence of rectal and sigmoid cancers in Black patients aged <40 years when compared to White patients of the same age (0.67 per 100,000, 95% CI, 0.60-0.74 vs. 0.51 per 100,000, 95% CI, 0.48-0.53) [8]. While these disparities result from a complex interplay of factors, reduced access to healthcare services and lower screening participation rates have frequently been implicated. Geographical disparities, often intertwined with SES and racial disparities, are another critical factor. Some studies showed that rural populations have higher CRC incidence rates and poorer outcomes than their urban counterparts, likely due to limited access to healthcare resources, lower screening rates, and differences in lifestyle factors [44].

Dietary/lifestyle modifications as prevention tools for mitigating the risk of eoCRC

The interplay of genetic and lifestyle elements contributes to the onset and prognosis of CRC. While genetic factors remain unmodifiable, lifestyle factors, such as obesity, physical inactivity, diet, smoking, and alcohol consumption, offer avenues for intervention to influence CRC incidence and prognosis, particularly in early-onset cases.

**Figure 2:**
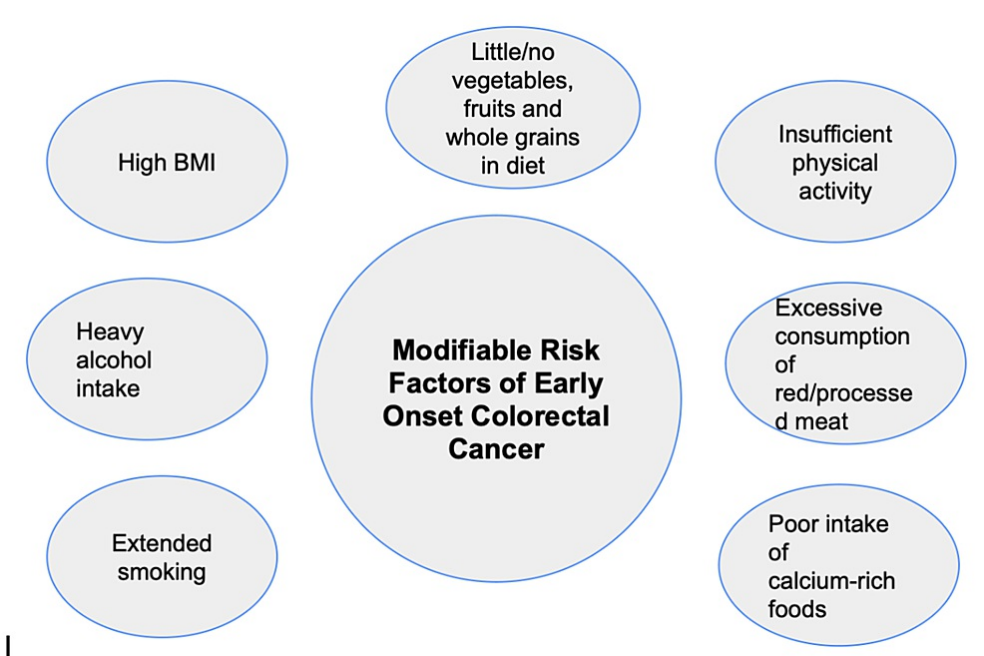
An original diagram showing modifiable risk factors of early-onset colorectal cancer.

A substantial body of research underscores the link between dietary choices and lifestyle factors in the evolution of CRC. In the United States, nearly half of CRC cases can be attributed to potentially modifiable risk factors, illustrated in Figure 2 below.

Dietary Factors

Fiber-rich foods and a Mediterranean diet: One of the key advantages of dietary fiber is that it promotes intestinal regularity. Whole grains, legumes, fruits, and vegetables supply insoluble fiber, which accelerates the passage of food and waste through the digestive system, reducing the time the colonic mucosa is exposed to possible carcinogens. Furthermore, the fermentation of fiber by beneficial intestinal bacteria produces short-chain fatty acids (SCFAs) such as acetate, propionate, and butyrate. SCFAs are notable for anti-inflammatory and anti-tumor effects, acting as a vital energy source for colonic cells and a regulator of cell differentiation and apoptosis, preventing malignant cell proliferation [30].

Fiber-rich foods are satiating and low in calories, which aids weight management. Given that obesity is a significant risk factor for CRC, the role of dietary fiber in weight regulation indirectly places it as a disease protector. The literature also suggests that high-fiber diets have the ability to reduce the colonic concentration of secondary bile acids, which, in excess, might cause damage to the colon lining and enhance carcinogenic pathways. Moreover, the anti-inflammatory effects of certain fibers, particularly those derived from whole grains, may provide an additional line of protection against CRC initiation and progression [30].

A substantive body of research links high consumption of red and processed meats with an increased CRC risk attributed to the carcinogenic compounds formed during cooking and preservation. Similarly, in the study by Jones et al., adhering to a Mediterranean diet was associated with a reduction in the risk of CRC, particularly rectal cancer, in women [30]. The protective effect of this diet is believed to be due to its high content of dietary fibers found in fruits, vegetables, nuts, and legumes, as well as its low content of red meat. These dietary fibers have the ability to dilute carcinogens in the feces, bind to carcinogenic bile acids, and decrease the acidity and time of colonic transit. Adopting a plant-based or Mediterranean-style diet, which emphasizes poultry, fish, legumes, and nuts, can mitigate these risks [30,32].

Micronutrients: Certain micronutrients, such as calcium and vitamin D, have demonstrated protective potential against CRC. Calcium may protect the colon by binding harmful bile acids and ionized fatty acids, controlling cellular proliferation, or triggering apoptosis at the intracellular level. A prospective study determined that higher concentrations of serum vitamin D are associated with lower incidences and improved survival of CRC, implying that vitamin D plays a role in the pathogenesis of eoCRC [45]. Although vitamin D regulates calcium absorption, there is growing evidence that it may also function through several biological calcium-independent routes, supporting or repressing certain cellular activities such as tumor genesis. Vitamin D participates in mitochondrial metabolism and may have anti-inflammatory properties. Furthermore, vitamin D may interfere with the metabolism of the colon microbiota. Without discounting the possibility of a synergistic/interaction between vitamin D and calcium, current evidence suggests that they may serve as independent preventive agents against CRC. Nevertheless, it is pivotal to seek natural dietary sources, given the equivocal benefits and possible risks associated with supplementation [46].

Dairy products, soy, and probiotics: In a meta-analysis conducted by Chapelle et al., it was discovered that the consumption of dietary products can help protect against the development of CRC with a risk reduction ranging from 0.81 to 8.87 [29]. Additionally, individuals who consume soy products showed a moderate decrease in their risk of CRC (RR = 0.85 to 0.92) compared to those who do not consume soy [2,29]. Therefore, increasing the daily intake of dairy products may have a protective effect against CRC.

Similarly, a growing body of evidence has established the link between gut microbiota, the onset of inflammation, and, ultimately, metabolic disorders and cancer [27]. Probiotics, such as SCFAs, have demonstrated potential in preventing CRC. SCFAs are metabolites of gut microbiota. They are a crucial source of energy for the cells of the large intestine, and they help maintain normal barrier function in the intestine. They have been found to stall cancer cell division and provide protection against both obesity and CRC. Additionally, other probiotics like *Akkermansia muciniphila* and *Lactobacillus* have also shown promise in preventing CRC [27]. Overall, incorporating probiotics into the diet, particularly those that produce SCFAs like *Akkermansia muciniphila* and *Lactobacillus*, may contribute to the prevention of CRC and support overall gut health.

Lifestyle Factors

Physical activity: Prolonged sedentary TV viewing has been linked with an increased risk of young-onset CRC, particularly of the rectum, emphasizing the importance of maintaining an active lifestyle [3]. Regular physical activity has been found to reduce obesity and systemic inflammation, a well-known risk factor for several cancers, including CRC. This anti-inflammatory effect is demonstrated by decreases in obesity-related inflammatory markers such as C-reactive protein (CRP), interleukin-6 (IL-6), zonulin (a marker of intestinal permeability), TMAO (cardiovascular disease-related microbial metabolite), and IL-1 (proinflammatory factor) and increased levels of IL-10 (anti-inflammatory factor) in non-obese individuals are individuals who are physically active [27]. Furthermore, physical activity enhances insulin sensitivity, resulting in lower circulating insulin levels. Elevated insulin and the presence of insulin-like growth factors (IGFs), notably IGF-1, have been linked to the development of CRC, as these molecules can accelerate colonic epithelial cell proliferation. Physical activity may directly lower the growth potential of precancerous lesions in the colon by improving insulin sensitivity.

The reduction of intestinal transit time is another way physical activity may lessen the incidence of eoCRC. Physical activity can accelerate waste transit through the colon, minimizing the colon's exposure to possible carcinogens found in feces. Furthermore, exercise has been proven to regulate the levels of various hormones, including estrogen, which indirectly impacts insulin and other growth factors. Although the relationship between estrogen and CRC is complicated, achieving hormonal balance through physical activity may help lower cancer risk. Furthermore, physical activity improves immune system function, allowing the body to identify and destroy developing cancer cells more effectively [25].

Physical activity, when combined with a good diet, can aid in maintaining a healthy weight. Obesity is a risk factor for CRC, and physical activity can indirectly lessen the risk by reducing obesity. Adipokines and other signaling chemicals produced by adipose (fat) tissue can increase inflammation and tumor growth.

Limiting alcohol and tobacco consumption: When consuming at least 14 drinks per week of alcohol, Rosato et al. discovered that the odds ratio for developing eoCRC was 1.56 [32]. Also, Khan et al., in their case-control study, found that smoking increased the risk of CRC by 2.1 times compared to healthy non-smokers (OR = 2.12, 95% CI: 1.08-4.17, P = 0.027) [34]. Meanwhile, the consumption of alcohol raised the CRC risk to 3.9 times (P < 0.001). Overall, individuals who indulged in any of these addictive substances faced a higher risk of CRC compared to those who abstained from using tobacco or alcohol (P = 0.003). They also asserted that even after quitting cigarette smoking, the risk of CRC continued to rise [34]. A cross-sectional analysis performed on 20-39-year-old participants in South Korea by Kim et al. revealed that in the 30-39 years age group, several factors were identified as independent risk factors for advanced CRC, including age (OR, 1.09; 95% CI, 1.06-1.13), smoking (OR, 1.30; 95% CI, 1.05-1.61), and alcohol intake (OR, 1.34; 95% CI, 1.10-1.63) [33]. The study also found that the sensitivity of smoking in detecting both overall and advanced CRC was 57.1% and 54.2%, respectively, in the 30-39 years age group [33].

**Figure 3:**
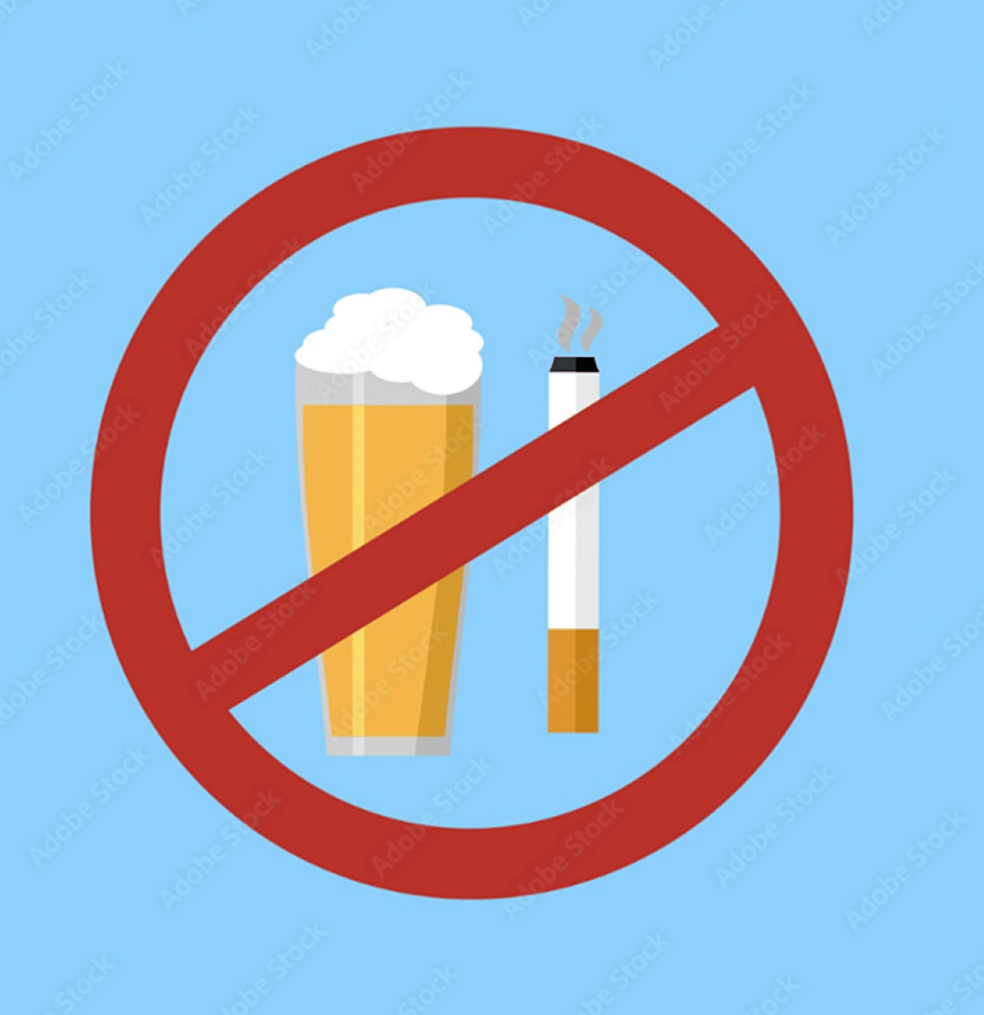
Illustration warning to stop alcohol and tobacco use.

Furthermore, the research highlighted that in individuals aged 30-39 years, smoking and alcohol intake were independent risk factors for both overall CRC and advanced CRC. Given that these modifiable factors are associated with an increased risk of advanced CRC in young adults under the age of 40, the study emphasized the importance of addressing these factors from a younger age as a crucial step in preventing CRC. It was also suggested that individuals with risk factors for advanced CRN may need to consider undergoing a screening colonoscopy before age 50 [33]. Figure 3 below, created by Adobe (Adobe Inc., San Jose, CA), is an illustration warning to stop alcohol and tobacco use.

Future directions and research gaps

It has been established that there has been a recent increase in the incidence of CRC in adolescents and young adults. At the same time, there is a drop in its incidence in older individuals. Brenner et al., after conducting a retrospective cohort study on the incidence of CRC in young adults in Canada, found that obesity was a primary culprit and proposed the need for further research. They also pointed out research gaps in the link between CRC in adolescents and young adults and too much folate in fortified foods, a sedentary lifestyle, the role of infectious agents like viruses, and overwhelming amounts of sodium and other food additives in refined foods [47].

Recent projections by You et al. suggest that by 2030, there will be a 90% increase in colon cancer and a 124% increase in new rectal cancer cases in adults aged 20-34 years. The authors attributed this to the fact that screening guidelines start at later ages, such that young adults with CRC present with symptoms and experience some delay before the commencement of treatment. The review also proposed a modification of risk factors and screening guidelines to reduce the incidence of CRC in adolescents and young adults [48].

Christodoulides et al. raised concern after a systematic review on sporadic CRC in patients < 49 years was conducted. They pointed out the need for detailed assessment of young adults, the commencement of screening at 40 years with rigid rectoscopy (as 2/3 of presenting young adults had cancer in the rectum), and targeted treatment options for affected young adults [49].

The future aim of CRC screening is to personalize the screening process according to an individual's risk for CRC. As guidelines become more advanced in predicting risk, they will also need to consider the possibility of uncertainty in predictions and make room for accommodating the patient's preferences. There is a need to find improved biomarkers or a combination, as current genetic risk information shows only slight enhancements in predicting risks. Additional research is needed to investigate genetic risk factors in populations other than non-Hispanic whites. Research should also focus on evaluating the adoption of the screening tools by patients and healthcare professionals and the actual screening results, such as adherence and findings, as discrepancies may occur [50].

With eoCRC, a subset of non-hereditary CRC, being more challenging, especially in patients < 35 years, with the worst survival rates in those < 30 years and a projection of a rise in its incidence, it is paramount that more clinical trials should be carried out with regards to targeted therapy for these patients [51].

## Conclusions

While overall rates of CRC in people over 50 years have decreased, the incidence of eoCRC is becoming alarming. Identifying the factors informing this trajectory for prophylaxis, optimal management, and individualized care delivery is paramount. Non-modifiable genetic conditions such as LS, FAP, MAP, and Peutz-Jeghers syndrome have been linked to the genesis of eoCRC. Similarly, a preponderance of research has shown the association of lifestyle factors like obesity, dietary habits, substance use, the built environment, and radiation with the development of CRC among individuals > 50 years. Encouraging individuals to make informed dietary and lifestyle choices and public health initiatives promoting these modifications can help reduce the burden of eoCRC. The disease course and survival rates of CRC depend on the time of detection, the stage of CRC at the time of diagnosis, and the "time to treatment" initiation. Hence, emphasis should be on increasing the awareness of eoCRC among practitioners and patients to increase the CRC suspicion index among symptomatic and asymptomatic younger patients, the availability and uptake of screening tests, and the discussion of preventive measures among patients > 50 years of age. Future studies should focus on personalizing the screening process according to an individual's risk for CRC.
